# Detection and Classification of Physiological Activation States Through Biosignal Analysis Using Machine Learning

**DOI:** 10.3390/s26206534

**Published:** 2026-10-15

**Authors:** Marco Leonelli, Jose Luis Jodra, Asier Salazar-Ramirez, Raquel Martinez Rodriguez, Javier E. Sanchez-Galan

**Affiliations:** 1 Facultad de Ciencias y Tecnología, Universidad Tecnológica de Panamá (UTP), Panama City 0819-07289, Panama; marco.leonelli@utp.ac.pa; 2 Department of Computer Architecture and Technology, University of the Basque Country (UPV/EHU), 20018 Donostia-San Sebastián, Spain; joseluis.jodra@ehu.eus; 3 Department of Systems Engineering and Automation, University of the Basque Country (UPV/EHU), 48940 Leioa, Spain; asier.salazar@ehu.eus; 4 Sistema Nacional de Investigación (SNI), SENACYT, Panama City 0816-02852, Panama; 5 Centro de Estudios Multidisciplinarios en Ciencias, Ingeniería y Tecnología (CEMCIT AIP), Panama City 0819-07289, Panama

**Keywords:** activation state classification, physiological arousal, biosignals, electrocardiogram, respiration, machine learning, deep learning, temporal convolutional network, transformer, hybrid architecture, heart rate variability, wearable devices, WESAD, affective computing

## Abstract

Monitoring autonomic nervous system responses offers significant potential for personalized health applications and early intervention. This study presents a machine learning framework for binary classification of physiological activation states using electrocardiogram (ECG) and respiratory (RESP) signals from the WESAD dataset. Stress and amusement were grouped as Activation, whereas baseline and meditation were grouped as Non-Activation. The processing pipeline incorporates R-peak detection, temporal segmentation, handcrafted feature extraction, and subject-independent evaluation. Conventional machine learning classifiers and deep learning architectures were evaluated, together with a proposed Hybrid Multi-Scale Temporal Convolutional–Conformer (MST-Conformer) combining temporal representations learned from ECG and RESP waveforms with handcrafted physiological features. Performance was assessed using 15-fold Leave-One-Subject-Out (LOSO) cross-validation. Adding respiration-derived features to ECG-derived features improved conventional machine learning performance, increasing the highest balanced accuracy from 0.752 to 0.807 and AUC from 0.802 to 0.886. The complete Hybrid MST-Conformer incorporating 27 physiological features achieved a mean accuracy of 0.825, balanced accuracy of 0.820, F1-score of 0.763, and AUC of 0.904. Subject-level analysis also revealed substantial physiological heterogeneity. These results support the potential of combining learned temporal representations with handcrafted physiological information for subject-independent physiological activation classification.

## 1. Introduction

Autonomic nervous system (ANS) responses reflect changes in physiological activity associated with cognitive, emotional, and environmental stimuli [1]. Objective and continuous monitoring of these responses has emerged as an important area of research at the intersection of biomedical signal processing, affective computing, and wearable healthcare [2,3]. While psychological stress is an important driver of autonomic activation, affective computing protocols frequently investigate physiological responses across different emotional and cognitive conditions [4].

Traditional psychological assessment relies on self-report questionnaires, clinical interviews, or observational scales. Although useful, these instruments are subjective, intermittent, and generally unsuited for continuous real-time monitoring [4]. Physiological biosignals provide an objective and continuous source of information about autonomic regulation. The electrocardiogram (ECG) and respiration (RESP) are particularly relevant modalities, as changes in autonomic activity can be reflected in cardiac rhythm, heart rate variability (HRV), respiratory rate, and respiratory dynamics [5,6]. In addition to autonomic measures, central nervous system markers such as electroencephalogram (EEG) signals have also demonstrated utility for physiological state detection [7].

Wearable sensor technology enables non-invasive and ambulatory acquisition of physiological signals [8]. Nevertheless, automated classification of physiological states remains challenging because physiological responses can vary substantially across individuals, complicating the development of models that generalize to previously unseen subjects [9]. Consequently, effective signal processing, physiological representation, and appropriate subject-independent validation strategies are important when developing models intended to generalize across individuals.

Classical machine learning approaches, including Linear Discriminant Analysis (LDA) [10], Support Vector Machine (SVM) [11], k-Nearest Neighbors (KNN), and Random Forest (RF) [12], have been widely applied to handcrafted cardiac and respiratory features [13,14]. Although these approaches are computationally efficient and can provide interpretable representations, their performance depends substantially on the quality and selection of the extracted features [15]. Deep learning methods provide an alternative by learning hierarchical representations directly from physiological time series [16,17]. One-dimensional convolutional neural networks (CNN1D) and multiscale architectures can extract localized temporal patterns [18,19,20], while recurrent architectures such as Long Short-Term Memory (LSTM) networks can capture sequential dependencies [21,22].

This study investigates the subject-independent binary classification of physiological activation states using ECG and RESP signals from the Wearable Stress and Affect Detection (WESAD) dataset [23]. The affective conditions available in WESAD were reorganized into two classes according to the classification framework adopted in this study: Activation, comprising stress and amusement, and Non-Activation, comprising baseline and meditation. Throughout this manuscript, the term “Activation” is therefore used to refer to the target class representing increased physiological activation rather than exclusively psychological stress.

A progressive experimental strategy was designed to investigate the contribution of different physiological modalities and representation approaches. First, handcrafted ECG-derived features were evaluated using conventional machine learning classifiers, followed by the incorporation of respiratory information. Deep learning architectures were then evaluated using the physiological signals, together with an analysis of inter-subject cardiac and respiratory variability. The Hybrid MST-Conformer was subsequently evaluated to investigate the complementary contribution of representations learned directly from the ECG and RESP signals and explicit physiological features. Finally, the effect of extending the physiological representation from a compact feature set to the complete set of available physiological features was investigated. All models were evaluated using a Leave-One-Subject-Out (LOSO) protocol to assess generalization to unseen subjects [24].

The remainder of this paper is organized as follows. Section 2 reviews previous studies on physiological state recognition and subject-independent classification. Section 3 describes the dataset, signal preprocessing, feature extraction, classification models, the Hybrid MST-Conformer architecture, and the experimental protocol. Section 4 presents the results of the four experiments, including the analysis of inter-subject physiological variability and the ablation studies. Section 5 discusses the main findings and their implications, and Section 6 summarizes the conclusions and outlines future research directions.

## 2. Related Work

### 2.1. Physiological Activation Detection

The ANS regulates physiological responses associated with cognitive and emotional states through the interaction of its sympathetic and parasympathetic branches [1]. HRV has been extensively studied as a non-invasive marker of autonomic regulation [5]. Time-domain, frequency-domain, and nonlinear HRV metrics have been investigated in relation to cognitive and emotional stress [13,25]. Respiratory activity is also closely related to autonomic regulation, providing complementary information about physiological state through changes in breathing dynamics [26]. Consequently, cardiac and respiratory signals constitute relevant sources of information for the characterization of physiological activation [4]. Beyond stress-related conditions, physiological signals have also been applied to the automatic detection of mental states associated with relaxation responses [27], illustrating their broader applicability to the characterization of affective and physiological states.

### 2.2. Machine Learning and Deep Learning in Affective Computing

Early approaches to affective computing largely relied on conventional machine learning techniques applied to handcrafted physiological features [2]. Algorithms such as SVM and RF have become established approaches for classification problems, including applications involving physiological and affective information [13]. Machine learning techniques have also been applied to the detection of other autonomic physiological responses [28]. However, advances in deep learning have enabled models to learn representations directly from physiological time series, reducing their dependence on exclusively handcrafted representations [17]. CNN architectures, initially developed primarily for image-related tasks [29], have subsequently been adapted to one-dimensional signals and time-series processing [18,19]. Multiscale approaches can further provide representations at different temporal resolutions, which is particularly relevant when physiological signals contain patterns occurring over different time scales [20].

### 2.3. Validation Strategies and Generalization

A major challenge in physiological signal classification is inter-subject variability, since physiological responses and their relationship with affective states can differ substantially across individuals [9]. Validation procedures in which samples from the same participant are included in both training and test sets may provide optimistic estimates of performance when the intended application requires generalization to previously unseen individuals. Subject-independent validation strategies are therefore particularly relevant in this context. LOSO cross-validation provides a framework in which each subject is held out for testing in turn, allowing model performance to be assessed on individuals whose data were not used for model training [24]. This evaluation strategy is consequently well suited to assessing subject-independent generalization in physiological classification problems.

### 2.4. Temporal Convolutional Networks and Transformer Architectures

TCNs provide an alternative to recurrent architectures for sequence modeling [30]. Through dilated convolutions and residual connections, TCNs can model temporal dependencies over increasingly large receptive fields while retaining the computational advantages of convolutional processing.

The Transformer architecture introduced self-attention mechanisms for modeling dependencies between elements of a sequence without relying on recurrent processing [31]. This ability to capture relationships across different positions in a sequence makes Transformer-based representations particularly relevant to temporal modeling.

Architectures combining convolutional processing and self-attention have subsequently been explored to integrate local and global information. The Conformer architecture, for example, combines convolutional modules with Transformer-based self-attention to capture complementary local and global dependencies [32]. This combination provides the conceptual basis for the Hybrid MST-Conformer investigated in this study, in which multi-scale temporal processing and Transformer-based representations are integrated for the analysis of physiological signals.

## 3. Materials and Methods

### 3.1. Dataset Presentation and Data Segmentation

The methodology was evaluated using the Wearable Stress and Affect Detection (WESAD) dataset, a publicly available multimodal database for affect and physiological arousal recognition. WESAD contains physiological recordings from 15 subjects, identified as S2–S17 (excluding S12). Participants were exposed to baseline, stress, amusement, and meditation protocols. In this study, chest-recorded electrocardiogram (ECG) and respiration (RESP) signals sampled at 700 Hz were utilized due to their high temporal resolution and direct coupling to autonomic nervous system activity.

Table 1 summarizes the duration of the whole recordings and Activation proportion for each participant, also including the intervals that were out of the interest of the present work.

For this work, the experimental conditions were mapped into a binary classification task: stress and amusement were grouped as the Activation class (label 1), while baseline and meditation were grouped as the Non-Activation class (label 0). This grouping was motivated by a dimensional representation of affect rather than by the assumption that stress and amusement constitute equivalent emotional states. According to the circumplex model of affect, affective states can be represented along two distinct dimensions, valence and arousal or activation [33]. Within this framework, states associated with physiological activation may differ in their affective valence. Therefore, the classification problem investigated in this study focuses specifically on the activation dimension, independently of differences in valence between the experimental conditions.

The class mapping was performed using a sliding-window approach with a window length of 20 s and a stride of 5 s. Each window was assigned the label corresponding to the majority class among all samples contained within that window. Consequently, windows were labeled as Activation (class 1) when the majority of samples belonged to either the stress or amusement protocols, and as Non-Activation (class 0) when the majority corresponded to either the baseline or meditation protocols. Windows whose majority label belonged to any other class were discarded and excluded from the study, as they corresponded to phases of the WESAD protocol that were not relevant to the objectives of this work.

Regarding the machine learning experimentation methodology used throughout the following sections, this work is based on the Leave-One-Subject-Out (LOSO) cross-validation evaluation strategy. In this approach, one subject from the dataset is held out for testing while all remaining subjects are used for training. This process is repeated iteratively until each subject has been used exactly once as the test subject. This strategy enables the full exploitation of the dataset for both training and validation purposes while providing a subject-independent evaluation of the machine learning algorithms.

It is important to note that subject-specific Z-score standardization was applied as a preprocessing step prior to the LOSO evaluation. Specifically, for each subject, the mean and standard deviation of every extracted feature were computed using all windows belonging to that subject, and these statistics were subsequently used to standardize only that subject’s feature vectors. After this preprocessing stage, the LOSO procedure was performed on the resulting normalized feature set. Consequently, the held-out test subject was represented using feature values standardized with its own statistics, whereas the training subjects were represented using their respective subject-specific statistics. Importantly, the normalization statistics of the test subject were used exclusively to standardize the samples of that same subject. They were never used to normalize training samples, nor were they involved in model training, feature selection, hyperparameter optimization, model selection, or any other stage of the learning process. Therefore, no information from the test subject influenced the training data or the model-fitting procedure.

Admittedly, in a real-world deployment scenario, the statistics required for standardizing the data of a previously unseen user would not be available beforehand. Nevertheless, practical affective computing systems of this kind may include a subject-specific calibration phase prior to operation. Such a calibration procedure may involve physiological data collected under different controlled conditions representative of the states that the system is intended to recognize, rather than being necessarily restricted to a resting baseline. These calibration data would provide the information required to estimate subject-specific normalization parameters and subsequently standardize new physiological samples consistently. Therefore, the subject-wise standardization adopted in this study was considered to represent a plausible calibration-based application scenario.

Under this experimental framework, all results presented in this study were obtained by applying the 15 LOSO folds available in the dataset. The training and testing partitions corresponding to each fold are summarized in Table 2.

### 3.2. Feature Extraction

This subsection describes the feature extraction process applied to the ECG and respiration signals. As mentioned previously, both signals were segmented into 20 s windows using a sliding-window strategy with a 5 s stride. The selected window length represents a trade-off between statistical robustness and temporal sensitivity [5]. On the one hand, it provides a sufficient number of samples to obtain reliable physiological measurements; on the other hand, it allows the detection of meaningful physiological changes, which generally occur over relatively long time scales rather than instantaneously.

The following subsubsections provide a detailed description of the features extracted from each physiological signal. Since different feature sets were considered throughout the study, the extracted features are presented separately for the corresponding machine learning experiments described later in this article. This organization allows the rationale and composition of each feature set to be clearly linked to the experimental results reported in their respective sections.

#### 3.2.1. Experiment 1: ECG Related Features

As will be described later, the first exploration of the classification problem addressed in this study focused exclusively on features derived from the ECG signal. The objective of this initial experiment was to assess the ability of cardiac-related physiological markers to discriminate between the proposed Activation and Non-Activation states.

Regarding the ECG signal, the first processing step consisted of detecting the R-peaks using the Pan-Tompkins algorithm [34]. Based on the detected beats, a total of 12 features were extracted for the initial experiment (described in Section 4.1). Three of these features are related to heart rate dynamics: the mean RR interval (RR_mean), the median RR interval (RR_med), and the mean heart rate (HR_mean).

In addition, five features commonly used in heart rate variability (HRV) analysis were computed: the standard deviation of RR intervals (RR_std), the Root Mean Square of Successive Differences (RR_rmssd), the percentage of consecutive RR intervals differing by more than 50 ms within the window (pNN50), the standard deviation of heart rate (HR_std), and the coefficient of variation of RR intervals (RR_cv), defined as the ratio between the standard deviation and the mean RR interval (RR_cv=RR_std/RR_mean).

Finally, the remaining four features were extracted to characterize the distribution and morphology of the RR interval series: the maximum and minimum RR intervals within the window (RR_max and RR_min), the range between the longest and shortest RR intervals (RR_range), and the IQR interquartile range of RR intervals (RR_iqr), defined as the difference between the 75th and 25th percentiles of the RR distribution. This latter feature provides a robust measure of cardiac variability that is less sensitive to outliers than range-based metrics.

#### 3.2.2. Experiment 2: ECG and Respiration Related Features

The second experiment (see Section 4.2) considered in this study extended the feature set used in the first experiment by incorporating information derived from the respiration signal, which resulted in a 22 feature space. To this end, a total of 10 respiration-based features were extracted from the respiration.

The first four features were frequency-domain descriptors corresponding to the normalized power of the respiration signal within the frequency bands of 0–0.45 Hz, 0.5–0.95 Hz, 1–1.45 Hz, and 1.5–2 Hz, denoted as *P_0_045*, *P_05_095*, *P_1_145*, and *P_15_2*, respectively.

In addition to these spectral features, four basic statistical descriptors were computed from the respiration signal: the mean respiratory amplitude (*RESP_mean*), the median respiratory amplitude (*RESP_med*), the range of the signal within the window, defined as the difference between its maximum and minimum values (*RESP_range*), and the standard deviation of the respiration signal (*RESP_std*).

Finally, two additional features were designed to characterize the similarity between the respiration signal and a set of pure sinusoidal reference signals. Specifically, the respiration signal was correlated with sinusoidal waves whose frequencies ranged from 0.01 Hz to 2 Hz in increments of 0.01 Hz. For each window, the maximum correlation coefficient obtained across all reference frequencies was determined, and the mean and standard deviation of these maximum correlation values were used as features, denoted as *CC_mean* and *CC_std*, respectively.

#### 3.2.3. Experiment 3: Handcrafted Features Selected for Deep Learning

The third experiment, described with more detail in Section 4.3, moved beyond the traditional machine learning approaches explored in the previous experiments and investigated whether deep learning architectures could achieve superior performance on the proposed classification task. To this end, four different deep learning models were evaluated: a one-dimensional convolutional neural network (CNN1D), a multi-scale one-dimensional convolutional neural network (CNN1D-MS), a bidirectional long short-term memory network (BiLSTM), and a Hybrid Multi-Scale Temporal Convolutional–Conformer (MST-Conformer) architecture.

From the feature extraction perspective, it was assumed that these models would be capable of automatically learning the most relevant representations from the ECG and respiration signals. Therefore, the CNN1D, BiLSTM, and MST-Conformer architectures received the standardized ECG and respiration signal segments corresponding to each analysis window as direct inputs. Similarly, the CNN1D-MS model received exactly the same standardized ECG and respiration signal segments as the CNN1D model. These signals were processed through three parallel convolutional branches with kernel sizes of 3, 7, and 15 samples, enabling the extraction of temporal patterns at multiple receptive-field scales.

Finally, the MST-Conformer was conceived as a hybrid architecture capable of combining the analysis of standardized raw signals with handcrafted physiological features. Consequently, in addition to the ECG and respiration signals, this model also received nine manually extracted features selected to provide a compact representation of the most relevant physiological information contained in both modalities.

Four of these features were already included in the feature sets employed in Experiments 1 and 2: *HR_mean*, *HR_std*, *RR_rmssd*, and *RESP_std*. The remaining five features were specifically incorporated for this model: ECG energy (*ECG_energy*), ECG variance (*ECG_variance*), ECG kurtosis (*ECG_kurtosis*), respiration variance (*RESP_variance*), and the mean breathing rate (*BR_mean*), computed as the average interval between consecutive respiratory peaks. Together, these features were intended to complement the representations automatically learned from the raw physiological signals by providing additional statistical and physiological information.

#### 3.2.4. Experiment 4: Union of All Features

No new features were extracted for the fourth experiment (Section 4.4). Instead, the proposed classification task was addressed using the complete feature set obtained from the previous experiments, including the 22 features considered in Experiment 2 (12 ECG-derived and 10 respiration-derived features) and the 5 additional features introduced in Experiment 3 (3 ECG-based and 2 respiration-based features). Therefore, the resulting feature space was composed of 27 physiological features in total. Furthermore, for the study of the hybrid MST-Conformed scenario, this experiment also included the standardized raw signals.

### 3.3. Classification Models

As mentioned previously, this work addresses the classification problem using both traditional machine learning and deep learning approaches. Concerning traditional techniques, four conventional machine learning classifiers were evaluated: LDA, SVM, KNN, and RF. LDA was considered as a linear baseline, whereas SVM with a radial basis function kernel was used to model nonlinear decision boundaries. KNN provided an instance-based classification approach, while RF represented an ensemble-based nonlinear classifier (further detail on the hyperparameters is given in Appendix B). Class weighting was applied to classifiers supporting this option to mitigate the effect of class imbalance. No threshold optimization procedure was performed for the conventional classifiers. Instead, predictions were obtained using their default decision rules. Specifically, LDA, KNN, and RF assigned the class with the highest posterior probability or majority vote, corresponding to an effective decision threshold of 0.5, whereas SVM predictions were based on the sign of the decision function, corresponding to a threshold of 0 on the distance to the separating hyperplane.

Deep learning models were also evaluated to learn discriminative temporal representations directly from the physiological signals. Three architectures were considered as reference deep learning models: CNN1D, CNN1D-MS, and BiLSTM. CNN1D used one-dimensional convolutional layers, nonlinear activations, pooling operations, and fully connected layers to extract temporal patterns from the ECG and RESP signals. BiLSTM was included as a recurrent architecture for bidirectional sequence modeling.

CNN1D-MS extended the convolutional approach by incorporating a multiscale representation learning strategy for the physiological signals. Instead of processing the input through a single convolutional pathway, three parallel convolutional branches with kernel sizes of 3, 7, and 15 samples were applied directly to the same standardized ECG and respiration segments. These branches enabled the extraction of signal patterns at different receptive-field scales while preserving the same input representation used by the CNN1D baseline. The resulting feature representations were subsequently concatenated and processed by a fully connected classification head.

Detailed layer specifications for CNN1D, BiLSTM, and CNN1D-MS are provided in Appendix A.1, Appendix A.2 and Appendix A.3, respectively. A summary of the main characteristics of these architectures is provided in Table 3.

### 3.4. Hybrid MST-Conformer Architecture

To combine automatically learned temporal representations with explicit physiological information, a Hybrid Multi-Scale Temporal Convolutional–Conformer (MST-Conformer) architecture was developed. The proposed model integrates two complementary information streams: (1) a raw-signal branch that learns temporal representations directly from the ECG and RESP waveforms, and (2) a handcrafted feature branch that incorporates cardiac and respiratory descriptors.

The raw-signal branch employs a Multi-Scale TCN consisting of three parallel temporal convolutional sub-networks with dilation-rate sets d∈{1,2,4}, {1,4,16}, and {1,8,64}. These branches capture temporal patterns at different receptive-field scales. Their outputs are concatenated along the channel dimension, yielding a representation of dimension $d_{\mathrm{model}}=3\times f_{\mathrm{TCN}}$, where $f_{\mathrm{TCN}}$ denotes the number of filters per branch. An adaptive average pooling layer reduces the temporal dimension to a fixed sequence length $L_{\mathrm{out}}$. Sinusoidal positional encoding is then applied before the resulting representation is processed by a Transformer encoder with *N* layers and multi-head self-attention [31]. Global average pooling over the temporal dimension produces the final representation of the raw-signal branch.

In its initial configuration, evaluated in Experiment 3, the feature branch receives the nine handcrafted physiological features described in Section 3.2.3. In Experiment 4, the same architecture is retained, but the input to the feature branch is expanded to the complete set of 27 physiological features described in Section 3.2.4. These features are standardized and processed by a two-layer MLP with batch normalization, ReLU activations, and dropout regularization.

**Figure 1:**
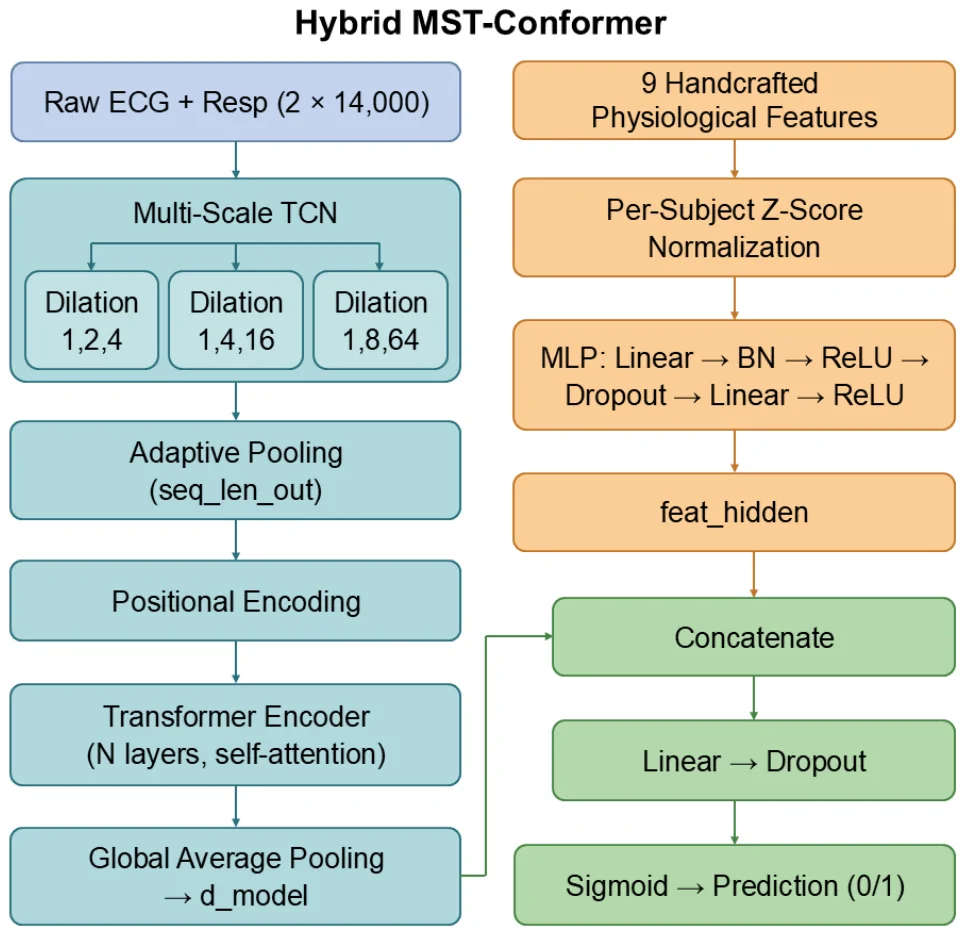
Architecture of the proposed Hybrid MST-Conformer. The raw-signal branch (**left**) processes ECG and RESP waveforms through a Multi-Scale TCN and Transformer encoder. The feature branch (**right**) processes nine handcrafted cardiac and respiratory features through an MLP. The representations obtained from both branches are concatenated for binary classification.

The representations obtained from the raw-signal and feature branches are concatenated and passed through a classification head consisting of a linear layer, dropout, and sigmoid activation to produce the binary classification output. The complete architecture is illustrated in Figure 1.

The model was implemented in PyTorch (version 2.5.1 with CUDA 12.4 support) [35] and trained using Focal Loss [36] combined with class weighting and label smoothing. It should be noted that the class-weighting term was not learnable. Instead, for each LOSO training fold, the positive-class weight α was computed from the class distribution of the training data as

(1) $$\alpha =1-r_{\mathrm{pos}},$$

where $r_{\mathrm{pos}}$ denotes the proportion of positive samples in the corresponding training fold. The resulting weighting factor remained fixed throughout optimization. The employed Focal Loss can be expressed as

(2) $$\mathrm{FL}\left(p_{t}\right)=-\alpha_{t}{\left(1-p_{t}\right)}^{\gamma }\;\mathrm{BCE}\left(y_{\mathrm{smooth}},p_{t}\right),$$

where $p_{t}$ denotes the predicted probability of the ground-truth class, γ is the focusing parameter, and $\alpha_{t}$ corresponds to the class-specific weighting factor. The modulating factor ${\left(1-p_{t}\right)}^{\gamma }$ progressively reduces the contribution of well-classified samples, enabling the network to focus on more difficult examples and improving robustness to class imbalance.

To further regularize training, label smoothing was applied prior to loss computation according to

(3) $$y_{\mathrm{smooth}}=\left(1-\lambda \right)y+0.5\lambda ,$$

where λ is the label-smoothing coefficient and *y* is the original binary label. This operation prevents the network from producing excessively confident predictions and improves generalization. Consequently, the combination of Focal Loss and label smoothing simultaneously addresses class imbalance, emphasizes difficult samples, and mitigates overfitting.

Concerning hyperparameter tuning, the hyperparameters were optimized using Optuna [37] with a Tree-structured Parzen Estimator (TPE) sampler over 25 trials. To reduce the computational burden associated with repeated optimization, the hyperparameter search was conducted only once for each architectural variant using a reduced search subset. Specifically, six subjects were selected for hyperparameter tuning, and each Optuna trial was evaluated over three LOSO folds generated from this subset. The optimization objective was defined as the average F1-score obtained across these folds. The hyperparameter configuration yielding the highest objective value was subsequently fixed and used for all folds of the final 15-fold LOSO evaluation. Although hyperparameter optimization and final model evaluation were conducted as separate computational stages, the six subjects included in the reduced optimization subset were subsequently also included among the test subjects of the final LOSO evaluation. Therefore, the hyperparameter-selection stage was not completely independent of all subjects used for final performance estimation.

The optimized hyperparameters included the number of TCN filters ($f_{\mathrm{TCN}}$), the number of Transformer layers (*N*), the number of attention heads, the feed-forward dimension, the feature-branch hidden size ($d_{\mathrm{feat}}$), dropout rate, learning rate, Focal Loss gamma, label smoothing, and weight decay (the values are specified in Appendix B). The OneCycleLR scheduler was used for learning-rate scheduling, and early stopping with a patience of 10 epochs was applied based on the validation F1-score.

### 3.5. Validation Strategy and Metrics

Model performance was evaluated using a 15-fold Leave-One-Subject-Out (LOSO) cross-validation strategy [24]. In each fold, all data from one participant were held out for testing, ensuring subject-independent evaluation and preventing samples from the test subject from being included in model training.

For the conventional machine learning models, the data from the remaining 14 subjects were used for model training. For the deep learning models, one of these 14 subjects, selected following a circular order, was reserved as a validation subject, while the remaining 13 subjects were used for training. The validation set was used for model selection procedures, including early stopping and classification-threshold optimization. Thus, each deep learning fold comprised 13 training subjects, one validation subject, and one test subject, as summarized in Table 2.

Model performance was assessed using accuracy, balanced accuracy, F1-score, area under the receiver operating characteristic curve (AUC), sensitivity, and specificity. Accuracy measures the overall proportion of correctly classified samples, whereas balanced accuracy accounts for class imbalance by averaging sensitivity and specificity. The F1-score, defined as the harmonic mean of precision and recall, provides a complementary measure of classification performance when the class distribution is imbalanced [38]. AUC evaluates the discriminative ability of the classifier across classification thresholds [39], while sensitivity and specificity quantify the ability to correctly identify Activation and Non-Activation windows, respectively.

Statistical comparisons between models were performed using the subject-level performance values obtained across the 15 LOSO folds as paired observations. For the comparison of the Hybrid MST-Conformer with the baseline deep learning architectures, paired *t*-tests and two-sided Wilcoxon signed-rank tests were performed, and Cohen’s *d* was used to quantify effect size. For the additional comparisons of the final complete Hybrid MST-Conformer with SVM, RF, and the FeaturesOnly configuration, two-sided Wilcoxon signed-rank tests were used. Statistical significance was assessed at p<0.05.

For the deep learning models, the classification threshold was selected independently in each LOSO fold using only the corresponding validation subject. The threshold maximizing balanced accuracy was selected from candidate values ranging from 0.05 to 0.95 and subsequently applied to the held-out test subject.

## 4. Results

This section presents the results obtained from the four experiments whose feature extraction was described in Section 3.2, following a progressive strategy aimed at evaluating the contribution of different sources of physiological information and classification approaches. All results are reported under the LOSO evaluation framework defined in Section 3.5.

### 4.1. Experiment 1: Conventional Machine Learning Results with ECG-Based Features

As a first approach to the classification problem, Experiment 1 evaluated whether information derived exclusively from the ECG signal was sufficient to discriminate between Activation and Non-Activation states. As described in Section 3.2.1, 12 ECG-derived features were used together with four conventional classifiers: LDA, SVM, KNN, and RF.

The results obtained under LOSO validation are presented in Table 4. Among the four classifiers evaluated, LDA achieved the highest mean balanced accuracy (0.752±0.105), F1-score (0.688±0.129), and AUC (0.802±0.122), together with an accuracy of 0.780±0.117. The highest sensitivity was obtained with KNN (0.684±0.109), whereas RF achieved the highest specificity (0.857±0.103).

Although these results indicate that ECG-derived features contain relevant discriminative information for distinguishing between Activation and Non-Activation states, the performance achieved, particularly in terms of balanced accuracy and F1-score, suggests that relying exclusively on cardiac information may limit classification performance. These findings provide the starting point for Experiment 2, in which additional physiological information is incorporated to evaluate whether a more comprehensive representation of the physiological response can improve discrimination performance.

### 4.2. Experiment 2: Conventional Machine Learning Results with ECG and Respiration Features

Following the results obtained in Experiment 1, Experiment 2 evaluated whether the incorporation of respiratory information could improve the discrimination between Activation and Non-Activation states. As described in Section 3.2.2, the ECG-derived feature set used in Experiment 1 was complemented with respiration-derived features, resulting in a total of 22 physiological features.

The results obtained with the four conventional classifiers are presented in Table 5. SVM achieved the highest mean accuracy (0.822±0.057), balanced accuracy (0.807±0.058), and F1-score (0.748±0.074). RF achieved the highest mean AUC (0.886±0.051) and specificity (0.862±0.067), whereas KNN obtained the highest sensitivity (0.760±0.062). LDA also showed competitive performance, with a balanced accuracy of 0.801±0.061 and an AUC of 0.886±0.058.

Compared with the results obtained using only ECG-derived features in Experiment 1, the incorporation of respiratory information improved the performance of the conventional classifiers. Considering the highest mean value obtained for each metric, accuracy increased from 0.780 to 0.822, balanced accuracy from 0.752 to 0.807, F1-score from 0.688 to 0.748, and AUC from 0.802 to 0.886. Sensitivity also increased from 0.684 to 0.760, while the highest specificity remained similar (0.857 in Experiment 1 and 0.862 in Experiment 2). Overall, these results suggest that incorporating respiration-derived features alongside cardiac information provides a more comprehensive physiological representation and improves the discrimination between Activation and Non-Activation states.

### 4.3. Experiment 3: Deep Learning Results

The results obtained in Experiments 1 and 2 showed that conventional machine learning methods can discriminate between Activation and Non-Activation states using features derived from ECG and respiratory signals. However, the performance achieved, particularly in terms of balanced accuracy and F1-score, suggests that further improvements in classification performance may be possible. Therefore, Experiment 3 explored the use of deep learning architectures capable of learning temporal representations directly from physiological signals.

Experiment 3 first compared different baseline deep learning architectures. The observed inter-subject variability was subsequently examined through a subject-level physiological analysis, followed by the evaluation of the proposed Hybrid MST-Conformer and the contribution of its different components through an ablation study.

#### 4.3.1. Initial Deep Learning Approach

In the first part of Experiment 3, three baseline deep learning architectures were compared: BiLSTM, CNN1D, and CNN1D-MS. The objective was to evaluate their performance in discriminating between Activation and Non-Activation states under subject-independent evaluation.

The results are presented in Table 6. CNN1D-MS achieved the highest mean balanced accuracy (0.580±0.048), F1-score (0.503±0.072), AUC (0.640±0.068), and sensitivity (0.692±0.238). In contrast, BiLSTM achieved the highest mean accuracy (0.549±0.111) and specificity (0.529±0.336). CNN1D achieved a balanced accuracy of 0.559±0.047 and an F1-score of 0.477±0.086.

Overall, the three baseline architectures showed substantial variability across LOSO folds, particularly in sensitivity and specificity. Since each fold corresponds to a different test subject, these results suggest relevant differences in subject-level classification performance. This variability motivated a more detailed analysis of individual physiological responses, which is presented in the following section.

#### 4.3.2. Inter-Subject Physiological Variability Analysis

The comparison among the deep learning architectures revealed considerable variability in performance across subjects under LOSO evaluation. To explore whether these differences could be related to individual physiological responses, heart rate (HR) and breathing rate (BR) were analyzed for each subject during Non-Activation and Activation states.

For each subject, mean HR and BR values were calculated for both conditions, together with the corresponding differences between Activation and Non-Activation (HR React and BR React), which were used as indicators of individual physiological reactivity. Table 7 presents these values together with the AUC obtained by CNN1D-MS for each subject. CNN1D-MS was selected for this analysis because, among the baseline deep learning architectures compared in the previous section, it achieved the highest mean balanced accuracy, F1-score, AUC, and sensitivity. AUC was used as the reference metric for the subject-level comparison because it provides a threshold-independent measure of the model’s discriminative ability.

The results show substantial inter-subject variability in both physiological levels and the magnitude of the response associated with the transition from Non-Activation to Activation. All subjects exhibited increases in both HR and BR during Activation, although the magnitude of these responses varied across subjects. Cardiac reactivity showed particularly large differences, ranging from +2.6 BPM in S2 to +43.6 BPM in S16. Respiratory reactivity varied within a narrower range, from +2.4 breaths/min in S2 to +5.6 breaths/min in S14.

**Figure 2:**
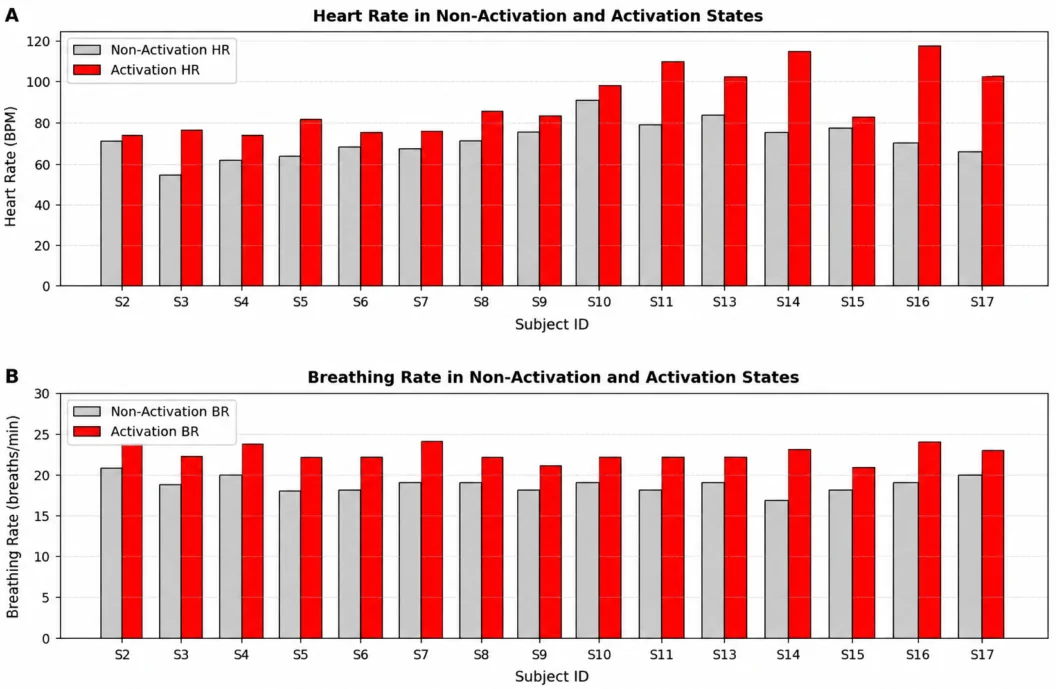
Heart rate (**A**) and breathing rate (**B**) per experimental condition across all 15 subjects.

Relevant differences were also observed in the physiological levels from which these responses occurred. For example, S10 exhibited the highest HR during Non-Activation (90.4 BPM), but showed a relatively small cardiac increase during Activation (+7.0 BPM). In contrast, S3 presented the lowest HR during Non-Activation (54.5 BPM) and a considerably larger cardiac response (+21.3 BPM). Differences were also observed in respiratory responses; for example, S14 showed the largest increase in BR (+5.6 breaths/min), whereas S2 showed the smallest (+2.4 breaths/min). These observations illustrate that inter-subject variability involves both the physiological levels observed during Non-Activation and the magnitude of the cardiac and respiratory responses associated with Activation. Figure 2 illustrates these differences in HR and BR across subjects and conditions.

When physiological reactivity was considered together with CNN1D-MS performance, a general tendency toward higher AUC values was observed in subjects with greater cardiac reactivity. The association between HR React and AUC was positive (Pearson r=0.563, p=0.029), whereas the association between BR React and AUC was weaker and did not reach statistical significance (Pearson r=0.381, p=0.161). However, this relationship was not consistent across all subjects. For example, S15 showed low cardiac reactivity (+4.6 BPM) and achieved an AUC of 0.690, whereas S8, with moderate cardiac reactivity (+13.7 BPM), achieved an AUC of 0.906. Therefore, the magnitude of cardiac reactivity may contribute to explaining part of the variability in subject-independent classification performance, but it does not, by itself, fully account for the model’s discriminative ability across subjects.

Subject S2 represents a particularly relevant case. This subject exhibits the lowest cardiac reactivity (+2.6 BPM) and an AUC of 0.226. Nevertheless, an AUC substantially below 0.5 should not be interpreted simply as an absence of discriminative information, since it indicates that the model tends to rank the two classes in the opposite direction to that expected. Consequently, the behavior observed for S2 suggests a particular generalization pattern that cannot be attributed exclusively to its low physiological reactivity.

**Figure 3:**
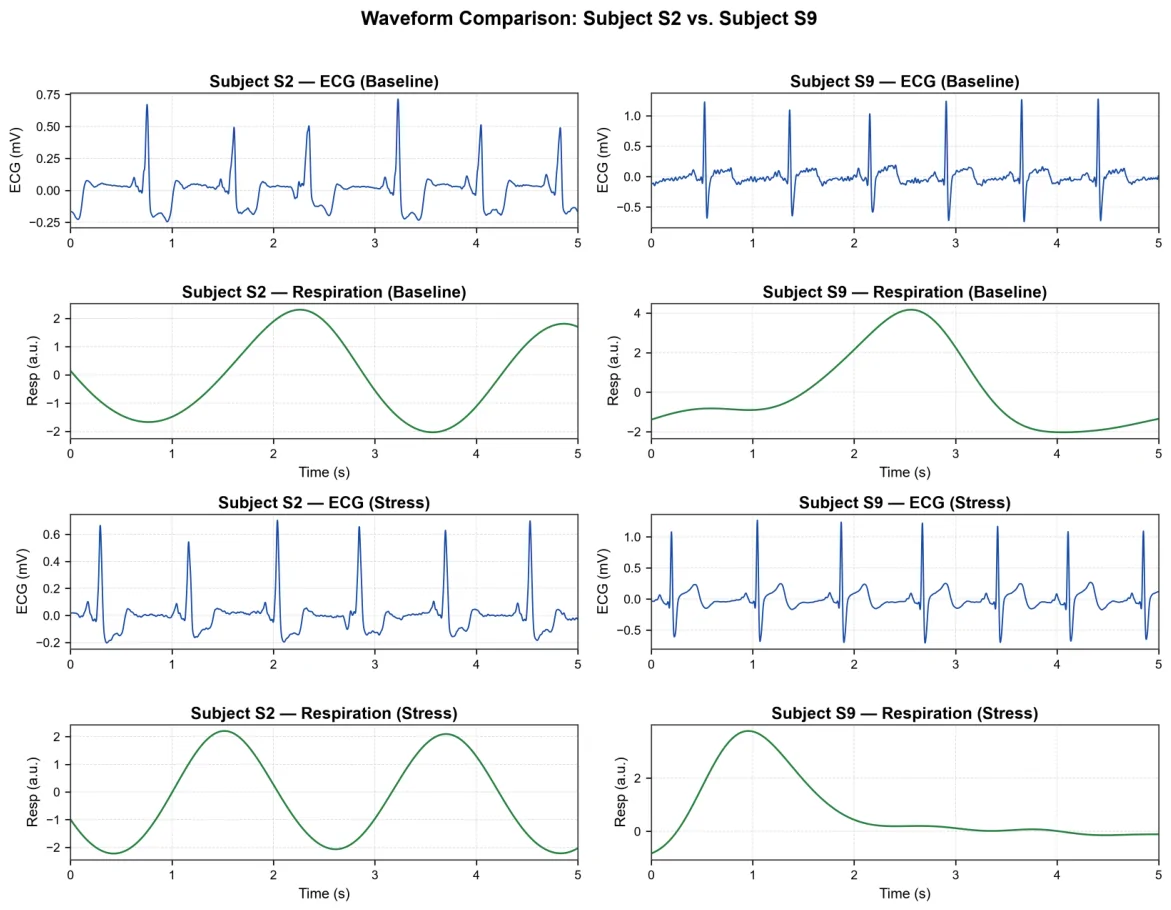
Representative raw ECG and respiration segments from subjects S2 (**left**) and S9 (**right**) during Non-Activation (**top**) and Activation (**bottom**) states. Differences between conditions are visually less apparent for S2, whereas S9 shows more noticeable changes in R–R interval spacing and respiratory waveform.

To further illustrate the inter-subject differences observed in the physiological signals, Figure 3 presents representative ECG and respiration segments from S2 and S9 during Non-Activation and Activation. Compared with S2, S9 shows more visually apparent differences between conditions in both R–R interval spacing and the respiratory waveform. These examples provide a qualitative illustration of the inter-subject physiological variability described above and should not be interpreted as a quantitative assessment of signal separability.

Overall, these results highlight substantial physiological heterogeneity across subjects, involving both individual physiological levels and differences in the magnitude of the response to Activation. This variability may contribute to the differences observed in subject-independent classification performance, although it does not provide a complete explanation of model behavior. The following section therefore evaluates the proposed Hybrid MST-Conformer, which combines temporal representations learned directly from the physiological signals with explicit physiological information.

#### 4.3.3. Hybrid MST-Conformer Evaluation and Ablation Study

The Hybrid MST-Conformer described in Section 3.4 was evaluated to investigate the complementary contribution of temporal representations learned directly from the ECG and RESP signals and explicit physiological information. In this experiment, the feature branch used the compact set of nine physiological features defined in Section 3.2.3.

Under the same LOSO protocol used for the deep learning architectures evaluated in Section 4.3.1, as shown in Table 8, the Hybrid MST-Conformer achieved an accuracy of 0.784±0.137, a balanced accuracy of 0.780±0.097, an F1-score of 0.729±0.108, and an AUC of 0.884±0.096. In terms of class-specific performance, the model achieved a sensitivity of 0.768±0.130 and a specificity of 0.792±0.241.

The analysis of the subject-level results shows that, although the Hybrid MST-Conformer improves AUC for most participants, substantial inter-subject variability remains. Consistent with the previous physiological analysis, a positive association between cardiac reactivity and model AUC was maintained (Pearson r = 0.534, *p* = 0.040), similar to that previously observed for CNN1D-MS (r = 0.563, *p* = 0.029). The case of S2 is particularly relevant, as this subject exhibited the lowest cardiac reactivity (+2.6 BPM) and achieved an AUC of 0.606 with the hybrid model. In contrast to the AUC of 0.226 obtained with CNN1D-MS, which reflected a tendency to rank the two classes in the opposite direction, the new result indicates limited discriminative ability and is more consistent with the low physiological reactivity observed in this subject.

The results achieved by the proposed MST-Conformer were higher than those obtained by the three baseline architectures for the main classification metrics. Taking CNN1D-MS as a reference, since it achieved the highest mean balanced accuracy, F1-score, AUC, and sensitivity among the baseline models in Section 4.3.1, balanced accuracy increased from 0.580 to 0.780, F1-score from 0.503 to 0.729, and AUC from 0.640 to 0.884. Accuracy also increased from 0.547 to 0.784 and specificity from 0.469 to 0.792, whereas the increase in sensitivity was more moderate, from 0.692 to 0.768.

To assess whether the observed differences were consistent across subjects, paired statistical comparisons were performed between the Hybrid MST-Conformer and each of the three baseline architectures using the results obtained across the 15 LOSO folds. As shown in Table 9, the differences in balanced accuracy, F1-score, AUC, and accuracy were statistically significant against all three models according to both the paired *t*-test and the Wilcoxon signed-rank test (p<0.001). Statistically significant differences in specificity were also observed in all three comparisons. In contrast, the differences in sensitivity did not reach statistical significance for any of the three baseline models.

The magnitude of the observed differences was also substantial for the main classification metrics. Cohen’s *d* indicated large effect sizes for balanced accuracy, F1-score, AUC, and accuracy across all three comparisons. The largest differences were observed against CNN1D, with effect sizes of d=2.357 for balanced accuracy and d=2.246 for AUC. In contrast, the smaller effect sizes obtained for sensitivity are consistent with the absence of statistically significant differences for this metric.

After evaluating the performance of the hybrid architecture against the baseline models, an ablation study was conducted to assess the contribution of the different information sources and the main components of the Hybrid MST-Conformer. Five additional configurations were evaluated: removal of the physiological feature branch (NoFeat), use of the physiological feature branch alone (FeaturesOnly), removal of the TCN (NoTCN), removal of the Transformer (NoTransformer), and replacement of the Multi-Scale TCN with a single-dilation TCN (Single-dilation TCN). All ablation configurations followed the same experimental pipeline used for the complete Hybrid MST-Conformer, with only the component under evaluation being modified or removed. Table 10 summarizes the mean and standard deviation obtained across the 15 LOSO folds for the complete architecture and the five ablation configurations.

The NoFeat and FeaturesOnly configurations were used to assess the contribution of the two information sources integrated into the hybrid architecture. Removing the physiological feature branch (NoFeat) reduced balanced accuracy from 0.780 to 0.701 and F1-score from 0.729 to 0.606, while AUC decreased from 0.884 to 0.851. In contrast, using only the physiological feature branch (FeaturesOnly) resulted in balanced accuracy, F1-score, and AUC values of 0.700, 0.610, and 0.797, respectively. Therefore, neither information source used independently achieved the performance obtained when both were combined in the complete architecture.

The remaining configurations were used to assess the contribution of the temporal-processing components. Removing the TCN (NoTCN) reduced balanced accuracy, F1-score, and AUC to 0.735, 0.641, and 0.830, respectively, although this configuration achieved the highest specificity among all the evaluated configurations (0.838). Removing the Transformer (NoTransformer) also resulted in lower performance than the complete architecture, with a balanced accuracy of 0.748, an F1-score of 0.681, and an AUC of 0.872.

Finally, replacing the Multi-Scale TCN with a single-dilation TCN resulted in an accuracy of 0.770±0.079, a balanced accuracy of 0.742±0.063, an F1-score of 0.657±0.114, and an AUC of 0.874±0.053. Although this configuration retained an AUC close to that obtained by the complete architecture (0.874 versus 0.884), it showed lower balanced accuracy, F1-score, and sensitivity, with sensitivity decreasing from 0.768 to 0.650. These results support the contribution of multi-scale temporal processing to the overall performance of the architecture.

Overall, the complete architecture achieved the highest mean accuracy, balanced accuracy, F1-score, AUC, and sensitivity among the evaluated deep learning configurations; the only exception was specificity, for which the highest value was obtained by NoTCN (0.838 versus 0.792). These results indicate that the performance of the Hybrid MST-Conformer benefits from combining representations learned directly from the ECG and RESP signals with explicit physiological information and different temporal-processing mechanisms. Nevertheless, despite the substantial improvement achieved by the hybrid architecture over the initial deep learning models, conventional machine learning approaches based on physiological features continued to show superior performance. In addition, the ablation analysis highlighted the contribution of the physiological feature branch. As part of the sequential exploratory development of the study, these observations motivated Experiment 4, in which this branch was expanded from the nine features used in Experiment 3 to the complete set of 27 available physiological features, with the aim of investigating whether a richer handcrafted representation could further improve the performance of the Hybrid MST-Conformer.

### 4.4. Experiment 4: Extended Feature Set and Hybrid MST-Conformer Results

As part of this sequential exploratory analysis, Experiment 4 investigated whether a more comprehensive representation of the available physiological information could further improve the classification of Activation and Non-Activation states. For this purpose, the complete set of 27 features obtained from the previous experiments was used, comprising the 22 features considered in Experiment 2 and the five additional features introduced in Experiment 3. This extended feature set was first evaluated using the four conventional classifiers and was subsequently incorporated into the feature branch of the Hybrid MST-Conformer.

The results obtained with the conventional classifiers are presented in Table 11. Compared with Experiment 2, in which 22 features were considered, the extended feature set produced modest improvements in several classification metrics. Among the conventional classifiers, RF achieved the highest mean accuracy (0.824±0.053), AUC (0.894±0.055), and specificity (0.863±0.063), whereas SVM achieved the highest balanced accuracy (0.811±0.061), F1-score (0.752±0.076), and sensitivity (0.768±0.078). Overall, these results indicate that the five additional physiological features provided a modest improvement in the performance of the conventional classifiers compared with the 22-feature representation evaluated in Experiment 2.

The complete set of 27 features was then incorporated into the feature branch of the Hybrid MST-Conformer, while retaining the branch that directly processes the ECG and respiration signals. To provide a more detailed view of the subject-wise performance of the final model, Table 12 presents the LOSO results obtained with the Complete Hybrid MST-Conformer using the 27-feature set, including the validation-derived classification threshold for each fold.

The results shown in Table 12 indicate that the validation-derived thresholds of the final 27-feature model had substantially lower variability across LOSO folds than those obtained with the nine-feature configuration reported in Table 8. Specifically, the threshold range decreased from 0.17–0.75 (0.471±0.125) to 0.35–0.55 (0.480±0.051). In addition, the thresholds of the final model were concentrated around the conventional 0.5 decision boundary. This reduced variability suggests a more stable decision boundary across subjects when the extended physiological feature set is incorporated into the complete architecture.

To further examine the potential heterogeneity introduced by grouping the original WESAD experimental conditions into the two binary classes, recall was additionally calculated separately for each protocol using the original WESAD protocol labels and the predictions of the final binary model. Specifically, the windows were separated according to their original WESAD experimental condition (baseline, meditation, stress, or amusement), prior to their grouping into Activation and Non-Activation. For the Non-Activation class, protocol-specific recall was 0.790 for baseline and 0.899 for meditation. For the Activation class, recall was 0.954 for stress and 0.485 for amusement. These results indicate that the individual experimental conditions grouped within the same binary class were not recognized equally by the classifier, with the largest difference observed between stress and amusement.

In addition, as done in Section 4.3.3, Table 13 summarizes the results obtained with the complete hybrid architecture and the five ablation configurations. The complete architecture achieved an accuracy of 0.825±0.061, a balanced accuracy of 0.820±0.056, an F1-score of 0.763±0.067, and an AUC of 0.904±0.053, with a sensitivity of 0.806±0.090 and a specificity of 0.834±0.093.

The ablation study further examined the contribution of the different information sources and architectural components using the extended physiological feature set. Removing the feature branch (NoFeat) resulted in the largest performance reduction, with a balanced accuracy of 0.578, an F1-score of 0.493, and an AUC of 0.612. In contrast, the FeaturesOnly configuration, which relied exclusively on the 27 physiological features, maintained a high classification performance, achieving a balanced accuracy of 0.808, an F1-score of 0.747, and an AUC of 0.901. Although these values remained slightly below those obtained with the complete architecture, they highlight the discriminative value of the extended physiological feature set. The paired comparison between the complete Hybrid MST-Conformer and the FeaturesOnly configuration showed no statistically significant differences for any of the six evaluation metrics (p>0.05 in all cases).

Modifications to the temporal-processing components resulted in smaller performance differences. The NoTCN configuration achieved a balanced accuracy of 0.811, an F1-score of 0.754, and an AUC of 0.897, whereas NoTransformer achieved values of 0.801, 0.743, and 0.898, respectively. The Single-dilation TCN configuration achieved a balanced accuracy of 0.809, an F1-score of 0.750, and an AUC of 0.902. Although these configurations approached the performance of the complete architecture for some metrics, the complete Hybrid MST-Conformer achieved the highest mean accuracy, balanced accuracy, F1-score, AUC, and sensitivity. Specificity was the only exception, with the highest value obtained by NoTCN (0.850±0.098 versus 0.834±0.093 for the complete architecture).

The comparison with Experiment 3 provides a direct assessment of the effect of expanding the physiological information used by the hybrid architecture. When the compact set of nine features was replaced by the complete set of 27 features, accuracy increased from 0.784 to 0.825, balanced accuracy from 0.780 to 0.820, F1-score from 0.729 to 0.763, and AUC from 0.884 to 0.904. Improvements were also observed in sensitivity, from 0.768 to 0.806, and specificity, from 0.792 to 0.834. Thus, the use of the complete physiological feature set resulted in higher mean values for all six evaluation metrics compared with the nine-feature Hybrid MST-Conformer evaluated in Experiment 3.

Compared with the conventional classifiers evaluated using the same 27-feature set, the complete Hybrid MST-Conformer achieved the highest mean accuracy (0.825±0.061, compared with 0.824±0.053 for RF), balanced accuracy (0.820±0.056, compared with 0.811±0.061 for SVM), F1-score (0.763±0.067, compared with 0.752±0.076 for SVM), AUC (0.904±0.053, compared with 0.894±0.055 for RF), and sensitivity (0.806±0.090, compared with 0.768±0.078 for SVM). In contrast, RF achieved the highest specificity (0.863±0.063, compared with 0.834±0.093 for the complete Hybrid MST-Conformer). However, despite the MST-Conformer got higher mean values in general, the paired comparisons across the 15 LOSO folds showed that the differences between the complete Hybrid MST-Conformer and SVM or RF were not statistically significant for any of the six evaluation metrics (p>0.05 in all cases).

The computational performance of the complete Hybrid MST-Conformer was also evaluated during inference. Inference times were measured for each LOSO test subject using an NVIDIA Tesla P40 GPU (NVIDIA Corporation, Santa Clara, CA, USA). Across the 15 test subjects, the model required an average of 2.189±0.623 s to classify a single 20-s ECG and respiration window. The time required to extract the handcrafted physiological features for each window was negligible compared with the model inference time. These measurements therefore provide an estimate of the computational cost associated with generating a classification once the corresponding physiological window is available.

Overall, the results of Experiment 4 show that expanding the physiological representation from nine to 27 features resulted in higher mean performance for the Hybrid MST-Conformer across all six evaluation metrics. The strong performance of the FeaturesOnly configuration also highlights the discriminative value of the extended physiological feature set. At the same time, the complete architecture achieved higher mean balanced accuracy, F1-score, AUC, and sensitivity than the FeaturesOnly configuration, showing that the combination of explicit physiological information and representations learned directly from the ECG and respiration signals yielded the highest mean performance for these metrics.

## 5. Discussion

The results of this study show that subject-independent classification of physiological activation states benefits from the integration of complementary physiological information and different representation strategies. The progressive experimental design allowed the contribution of ECG-derived information, respiration-related information, temporal representations learned directly from physiological signals, and explicit physiological features to be systematically evaluated while maintaining a consistent LOSO validation protocol throughout all experiments.

The first two experiments demonstrated the value of incorporating complementary physiological modalities. Using only ECG-derived features, the best conventional classification results included a balanced accuracy of 0.752 and an AUC of 0.802. After incorporating respiration-derived features, the highest values for these metrics increased to 0.807 and 0.886, respectively. Improvements were also observed in F1-score and sensitivity, which increased from 0.688 to 0.748 and from 0.684 to 0.760, respectively. These results suggest that respiratory dynamics provide information that complements the cardiac response when distinguishing between Activation and Non-Activation states. This finding supports the use of multimodal physiological information rather than relying exclusively on cardiac dynamics.

The deep learning experiments revealed an additional challenge associated with subject-independent classification: considerable variability in performance across subjects. The physiological analysis conducted in Experiment 3 showed that participants also differed substantially in their cardiac and respiratory responses between Activation and Non-Activation states. Although these differences cannot fully explain the variability in classification performance, they highlight the heterogeneity that a subject-independent model must accommodate and motivated the integration of explicit physiological information with representations learned directly from the signals.

The analysis of physiological reactivity provides additional insight into this inter-subject variability. In particular, a significant positive association was observed between the increase in heart rate from Non-Activation to Activation and the AUC obtained with CNN1D-MS (r=0.563, p=0.029), and a similar association was maintained with the Hybrid MST-Conformer (r=0.534, p=0.040). These findings suggest that subjects exhibiting greater cardiac responses between the two physiological states tend to present more discriminative patterns for subject-independent classification. However, this relationship was not consistent across all participants, and respiratory reactivity was not significantly associated with classification performance. Therefore, physiological reactivity may contribute to explaining part of the observed inter-subject variability, but it does not provide a complete explanation of the models’ discriminative ability.

Within this context, the Hybrid MST-Conformer achieved substantially higher performance than the three baseline deep learning architectures. Compared with CNN1D-MS, which achieved the highest mean balanced accuracy, F1-score, AUC, and sensitivity among the baseline architectures, the hybrid model increased balanced accuracy from 0.580 to 0.780, F1-score from 0.503 to 0.729, and AUC from 0.640 to 0.884. The paired comparisons across the 15 LOSO folds showed statistically significant differences in balanced accuracy, F1-score, AUC, and accuracy against all three baseline architectures. In contrast, sensitivity did not differ significantly, suggesting that an important contribution of the hybrid approach was not simply an increased detection of Activation states, but a more balanced discrimination between the two classes.

The ablation analysis in Experiment 3 further supported the complementary role of the two representation strategies. Neither the signal-based representation without the physiological feature branch nor the physiological feature branch used alone achieved the performance of the complete architecture. Moreover, removing or simplifying the temporal-processing components generally reduced the main classification metrics. These findings suggest that the performance of the Hybrid MST-Conformer cannot be attributed to a single component, but rather to the integration of learned temporal representations, explicit physiological information, and temporal-processing mechanisms.

Experiment 4 further investigated the role of explicit physiological information by expanding the feature branch from the compact set of nine features used in Experiment 3 to the complete set of 27 available features. This extension resulted in higher mean values for all six evaluation metrics of the complete Hybrid MST-Conformer. Balanced accuracy increased from 0.780 to 0.820, F1-score from 0.729 to 0.763, and AUC from 0.884 to 0.904, while sensitivity increased from 0.768 to 0.806. These results indicate that the more comprehensive physiological representation provided additional discriminative information beyond that contained in the compact feature set.

The relevance of the extended physiological representation was also evident in the conventional machine learning models and in the ablation analysis. Among the conventional classifiers, SVM achieved a balanced accuracy of 0.811, whereas RF achieved the highest AUC (0.894) and specificity (0.863). However, the complete Hybrid MST-Conformer achieved higher balanced accuracy (0.820), F1-score (0.763), AUC (0.904), and sensitivity (0.806) than the best values obtained by the conventional classifiers for these metrics. In addition, the FeaturesOnly configuration achieved a balanced accuracy of 0.808 and an AUC of 0.901, approaching the performance of the complete architecture. This finding highlights the strong discriminative value of the extended physiological feature set. However, the paired comparisons across the 15 LOSO folds showed that these differences were not statistically significant for any of the evaluated metrics when the complete Hybrid MST-Conformer was compared with SVM, RF, or FeaturesOnly. Therefore, the higher mean performance observed for the complete architecture should be interpreted as a descriptive improvement rather than as evidence of statistically significant superiority over these closely performing alternatives.

It should also be noted that some of the handcrafted ECG features included in the hybrid model, particularly ECG energy, variance, and kurtosis, are amplitude- and morphology-dependent descriptors. Unlike HRV-derived measures, these features may be influenced by inter-subject differences in ECG amplitude, electrode placement, skin–electrode contact, and acquisition conditions. Although per-subject standardization reduces baseline scaling differences, these descriptors should not be regarded as inherently cross-subject invariant. Their contribution should therefore be interpreted as complementary signal-level information rather than as purely physiological markers.

Interestingly, the differences between the complete architecture and some of the temporal ablations became relatively small when the complete physiological feature set was available. The NoTCN, NoTransformer, and Single-dilation TCN configurations achieved AUC values of 0.897, 0.898, and 0.902, respectively, compared with 0.904 for the complete architecture. This suggests that, when a rich physiological representation is explicitly provided to the model, the relative contribution of individual temporal-processing components may become less pronounced. Nevertheless, the complete architecture maintained the highest mean balanced accuracy, F1-score, AUC, and sensitivity, indicating that the learned signal representations still provide complementary information to the handcrafted physiological features.

Table 14 provides additional context by comparing the proposed framework with previously reported results on the WESAD dataset. These numerical comparisons should be interpreted with caution because the approaches differ in the physiological modalities used and in the definition of the classification task. In particular, the original WESAD benchmark by Schmidt et al. [23] uses the complete set of chest modalities, including ECG, EDA, EMG, respiration, and temperature, whereas the present study is restricted to ECG and respiration. Furthermore, the present work addresses the distinction between Activation and Non-Activation, grouping stress and amusement as Activation and baseline and meditation as Non-Activation. On the other hand, amusement was considered to be Non-Stress in [23], i.e., class 0, which is comprehensible as it is the opposite to stress. However, this distinction makes it a totally different classification problem.

As shown in Table 14, the original WESAD benchmark reports higher accuracy and F1-score for binary classification when the complete set of chest-recorded physiological modalities is available. However, the comparison is not direct because both the available physiological information and the class definitions differ from those considered in the present study. Within the ECG- and respiration-based framework evaluated here, the Hybrid MST-Conformer achieved higher mean F1-score and AUC than the conventional RF model and the baseline deep learning architectures. In particular, the final Hybrid MST-Conformer achieved an accuracy of 0.825, an F1-score of 0.763, and an AUC of 0.904. These results indicate that combining explicit physiological features with learned temporal representations provides an effective strategy for subject-independent physiological activation classification when the input is restricted to ECG and respiration.

The more recent studies included in Table 14 extend this comparison by considering different combinations of physiological signals and classification tasks. Bobade and Vani [40] employed a multimodal approach including ECG and respiration, together with other physiological signals, and reported an accuracy of 0.952 for Stress versus Non-Stress classification. Prajod and André [41], using ECG and HRV features, achieved an accuracy of 0.908 and an F1-score of 0.857 for the discrimination between Stress and Non-Stress. Yang et al. [42] demonstrated that respiration alone can provide relevant discriminative information, reporting an accuracy and F1-score of approximately 0.923 for distinguishing among Normal, Stress, and Meditation states. These results further support the relevance of ECG and respiration for the characterization of physiological states; however, differences in the physiological signals considered and, particularly, in the definition of the classification tasks prevent a direct comparison of these performance metrics with those obtained in the present study.

In light of the results obtained in this study, further improvements could potentially be achieved by incorporating additional sources of physiological information. Previous studies using WESAD, including the original work by Schmidt et al. [23], have considered a broader set of physiological modalities than the ECG and respiration signals used in the present study. Therefore, additional signals available in WESAD, such as electrodermal activity (EDA) or electromyography (EMG), could provide complementary information for characterizing physiological activation. In particular, EDA reflects sympathetic sudomotor activity through changes in skin conductance and may provide an additional physiological marker for subjects such as S2, who exhibited particularly low cardiac reactivity in the present analysis. Nevertheless, WESAD contains only 15 subjects and therefore represents a relatively limited population for assessing subject-independent generalization. Although WESAD was selected after considering other publicly available datasets, including the SWELL Knowledge Work Dataset [43], the Arachnophobia Dataset [44], CEBSDB [45], and DEAP [46], validation on independent datasets and larger and more diverse populations is required to assess the generalizability of the proposed framework.

The binary grouping of the original WESAD experimental conditions should also be considered when interpreting the results. Stress and amusement were jointly considered as Activation, whereas baseline and meditation were grouped as Non-Activation. As discussed in the methodology, this grouping was motivated by a dimensional representation of affect, in which arousal or activation and valence represent distinct dimensions [33], rather than by the assumption that the individual protocols represent equivalent emotional or physiological states. In this context, previous experimental research has shown that amusement, despite its positive valence, can be associated with elevated levels of arousal [47]. The protocol-specific analysis further supports this distinction. While baseline and meditation achieved recall values of 0.790 and 0.899, respectively, a considerably larger difference was observed within the Activation class, with recall values of 0.954 for stress and 0.485 for amusement. These findings reveal substantial intra-class heterogeneity, particularly within the Activation class, and indicate that grouping experimental conditions according to the activation dimension does not imply equivalent ECG and respiratory patterns or equivalent classification performance. A more detailed characterization of the physiological differences among the original WESAD conditions, as well as the evaluation of alternative classification settings, remains an important direction for future work.

The subject-specific normalization procedure also deserves consideration when interpreting the results of this study. For each participant, normalization statistics were estimated from all available windows, including those corresponding to the different experimental conditions. This approach is consistent with a calibration scenario in which physiological data representative of different states are collected from a new user before the system is used. In other application settings, however, calibration may be limited to an initial resting baseline. Under such conditions, the normalization parameters available during deployment would not be equivalent to those considered here. The results reported in this work should therefore be interpreted in the context of the adopted calibration procedure, while their applicability to baseline-only calibration remains to be established. Future studies could specifically evaluate the effect of estimating subject-specific normalization parameters exclusively from baseline recordings.

Another methodological limitation concerns the sequential model-development process and hyperparameter optimization. The study followed an exploratory experimental strategy in which observations obtained from preceding experiments informed subsequent analyses; in particular, the extended feature configuration evaluated in Experiment 4 was introduced after examining the results of Experiment 3. In addition, the six subjects used in the reduced Optuna hyperparameter optimization subset were subsequently also included among the test subjects of the final 15-fold LOSO evaluation. Although the held-out test subject in each final LOSO fold was excluded from model training, the architecture and hyperparameter-selection processes were therefore not completely independent of all subjects used for final performance estimation. Consequently, some degree of optimistic selection bias cannot be ruled out, and the reported results should be interpreted considering this methodological constraint. A stricter evaluation would require nested subject-wise cross-validation, in which architecture selection, feature-set selection, and hyperparameter optimization are performed exclusively using the training subjects of each outer LOSO fold. Future work will investigate this nested validation strategy to provide a fully independent estimate of subject-level generalization.

From a computational perspective, the mean inference time of 2.189±0.623 s per 20-s window obtained using an NVIDIA Tesla P40 GPU indicates that the computational cost of the Hybrid MST-Conformer is compatible with real-time classification when GPU resources of similar capability are available. This would make it feasible to deploy the model in a platform in which physiological data are transmitted to a GPU-enabled processing system and a classification response is generated once each analysis window becomes available. However, these inference times were obtained using a dedicated GPU and should not be extrapolated to resource-constrained embedded hardware. Direct execution on an embedded wearable platform with substantially lower computational capabilities may result in considerably longer inference times and would require further evaluation and potentially model optimization, compression, or hardware-specific acceleration.

## 6. Conclusions

This study investigated the subject-independent classification of Activation and Non-Activation states using ECG and respiratory signals through conventional machine learning methods and deep learning architectures. A progressive experimental strategy was followed to assess the contribution of different physiological modalities, physiological feature representations, and model components under a LOSO validation protocol.

The results showed that respiratory information complements cardiac information and that inter-subject physiological variability represents an important challenge for subject-independent classification. The use of the complete set of 27 physiological features provided a richer representation of the available physiological information and resulted in higher mean performance for the Hybrid MST-Conformer than the compact nine-feature configuration evaluated in Experiment 3. Conventional machine learning methods also achieved high performance with the extended feature set, highlighting the discriminative value of an appropriate physiological representation.

The proposed Hybrid MST-Conformer combined temporal representations learned directly from ECG and respiratory signals with handcrafted physiological features. Using the complete set of 27 features, the complete architecture achieved an accuracy of 0.825, a balanced accuracy of 0.820, an F1-score of 0.763, and an AUC of 0.904, with a sensitivity of 0.806 and a specificity of 0.834. The ablation studies supported the complementary contribution of explicit physiological information and representations learned directly from the signals, while also showing that the relative contribution of individual architectural components may depend on the richness of the physiological feature representation.

Overall, these findings support the integration of explicit physiological information and temporal representation learning for the subject-independent classification of physiological activation states. Future studies should validate the proposed approach on independent datasets, larger and more diverse populations, and less controlled experimental conditions to further assess its generalizability and potential application to wearable physiological monitoring systems.

## Figures and Tables

**Table 1:** Characteristics of the WESAD dataset used in this work.

Subject	Samples	Duration (min)	Activation (%)
S2	4,255,300	101.3	29.0
S3	4,545,100	108.2	29.7
S4	4,496,100	107.1	29.3
S5	4,380,600	104.3	29.1
S6	4,949,700	117.9	29.5
S7	3,666,600	87.3	29.1
S8	3,826,200	91.1	30.3
S9	3,656,100	87.1	29.4
S10	3,847,200	91.6	31.8
S11	3,663,100	87.2	30.5
S13	3,875,900	92.3	29.8
S14	3,883,600	92.5	30.3
S15	3,676,400	87.5	30.7
S16	3,941,700	93.9	30.3
S17	4,144,000	98.7	31.8

**Table 2:** LOSO fold sample distribution. Each fold reserves one subject for testing and the next (in circular order) for validation; the remaining 13 subjects form the training set.

Fold	Test Subject	Test Windows	Val Subject	Val Windows	Train Subjects	Train Windows
1	S2	578	S3	587	13	7823
2	S3	587	S4	592	13	7812
3	S4	592	S5	602	13	7797
4	S5	602	S6	598	13	7791
5	S6	598	S7	597	13	7796
6	S7	597	S8	601	13	7793
7	S8	601	S9	599	13	7791
8	S9	599	S10	615	13	7777
9	S10	615	S11	604	13	7772
10	S11	604	S13	603	13	7784
11	S13	603	S14	604	13	7784
12	S14	604	S15	606	13	7781
13	S15	606	S16	603	13	7782
14	S16	603	S17	602	13	7786
15	S17	602	S2	578	13	7811
Total		8991				

Total class distribution across all subjects: Non-Activation = 5883 windows (65.4%), Activation = 3108 windows (34.6%). Each subject is tested exactly once across the 15 folds.

**Table 3:** Summary of the deep learning architectures, including parameter counts, model size, and key design characteristics.

Architecture	Total Parameters	Trainable Params	Model Size (MB)	Key Characteristics
CNN1D	60,513	60,513	0.23	Sequential convolutions, single temporal scale
BiLSTM	47,521	47,521	0.18	Bidirectional sequence modeling
CNN1D-MS	284,321	284,321	1.08	Multiscale parallel convolutional branches
Input Configuration:2 channels (ECG, RESP) × 14,000 temporal steps = (14,000, 2)				

Model sizes are approximate and assume float32 precision. All models produce a single binary output through a sigmoid activation function.

**Table 4:** LOSO classification results obtained from 12 handcrafted ECG features (mean ± std across 15 folds). The best mean value for each evaluation metric is shown in bold.

Model	Accuracy	Bal. Acc.	F1	AUC	Sensitivity	Specificity
LDA	0.780±0.117	0.752±0.105	0.688±0.129	0.802±0.122	0.664±0.087	0.841±0.153
SVM	0.766±0.109	0.731±0.111	0.650±0.149	0.773±0.125	0.618±0.130	0.844±0.116
KNN	0.707±0.086	0.702±0.089	0.619±0.105	0.762±0.111	0.684±0.109	0.719±0.085
RF	0.780±0.102	0.745±0.104	0.669±0.144	0.783±0.121	0.632±0.121	0.857±0.103

Bal. Acc.: Balanced Accuracy; Sensitivity: True Positive Rate (Activation); Specificity: True Negative Rate (Non-Activation).

**Table 5:** LOSO classification results obtained from 22 handcrafted ECG and respiration features (mean ± std across 15 folds). The best mean value for each evaluation metric is shown in bold.

Model	Accuracy	Bal. Acc.	F1-Score	AUC	Sensitivity	Specificity
LDA	0.816±0.065	0.801±0.061	0.742±0.076	0.886±0.058	0.754±0.064	0.848±0.083
SVM	0.822±0.057	0.807±0.058	0.748±0.074	0.883±0.065	0.759±0.071	0.855±0.057
KNN	0.778±0.045	0.774±0.046	0.704±0.056	0.841±0.053	0.760±0.062	0.788±0.049
RF	0.820±0.053	0.802±0.050	0.743±0.067	0.886±0.051	0.742±0.061	0.862±0.067

Bal. Acc.: Balanced Accuracy; Sensitivity: True Positive Rate (Activation); Specificity: True Negative Rate (Non-Activation).

**Table 6:** LOSO classification performance of the baseline deep learning architectures (mean ± std across 15 folds). The best mean value for each evaluation metric is shown in bold.

Model	Bal. Acc.	F1-Score	AUC	Accuracy	Sensitivity	Specificity
BiLSTM	0.558±0.058	0.425±0.165	0.635±0.095	0.549±0.111	0.586±0.354	0.529±0.336
CNN1D-MS	0.580±0.048	0.503±0.072	0.640±0.068	0.547±0.090	0.692±0.238	0.469±0.244
CNN1D	0.559±0.047	0.477±0.086	0.622±0.082	0.528±0.081	0.665±0.256	0.454±0.240

Bal. Acc.: Balanced Accuracy; Sensitivity: True Positive Rate (Activation); Specificity: True Negative Rate (Non-Activation).

**Table 7:** Per-subject physiological responses during Non-Activation and Activation states. HR React and BR React represent the changes in heart rate and breathing rate, respectively, between Activation and Non-Activation states. Subjects are sorted by CNN1D-MS AUC in ascending order.

Subject	HR No-Activat. (BPM)	HR Activation (BPM)	HR React (BPM)	BR No-Activat. (br/min)	BR Activation (br/min)	BR React (br/min)	CNN1D-MS AUC
S2	71.1	73.7	+2.6	21.1	23.5	+2.4	0.226
S10	90.4	97.4	+7.0	18.6	21.9	+3.3	0.531
S15	77.8	82.4	+4.6	17.7	20.8	+3.1	0.690
S6	68.7	74.5	+5.8	17.9	22.0	+4.1	0.756
S11	79.2	107.3	+28.1	17.8	22.0	+4.2	0.756
S9	75.5	82.6	+7.1	18.3	21.0	+2.7	0.774
S7	67.5	75.7	+8.2	19.0	23.8	+4.7	0.784
S13	83.2	100.9	+17.7	19.2	22.4	+3.2	0.789
S5	63.5	81.5	+18.0	18.0	21.5	+3.6	0.792
S4	61.8	73.6	+11.8	20.1	23.9	+3.8	0.801
S14	75.0	111.6	+36.6	17.4	23.0	+5.6	0.825
S3	54.5	75.7	+21.3	18.5	22.3	+3.8	0.845
S17	65.8	101.2	+35.4	19.7	22.6	+2.9	0.874
S8	70.9	84.6	+13.7	19.3	21.9	+2.6	0.906
S16	69.9	113.4	+43.6	19.4	23.9	+4.5	0.910

HR: Heart Rate; BR: Breathing Rate; BPM: beats per minute; br/min: breaths per minute.

**Table 8:** Subject-wise LOSO classification results obtained with the complete Hybrid MST-Conformer using the nine handcrafted physiological features.

Subject	Threshold	Accuracy	Bal. Acc.	F1	AUC	Sensitivity	Specificity
S2	0.35	0.452	0.583	0.550	0.606	0.990	0.175
S3	0.45	0.905	0.902	0.866	0.965	0.892	0.912
S4	0.34	0.809	0.749	0.667	0.900	0.562	0.936
S5	0.47	0.861	0.811	0.761	0.932	0.657	0.965
S6	0.56	0.886	0.857	0.821	0.938	0.765	0.949
S7	0.48	0.789	0.752	0.672	0.809	0.639	0.866
S8	0.42	0.604	0.696	0.635	0.945	0.995	0.397
S9	0.75	0.877	0.825	0.785	0.950	0.662	0.987
S10	0.60	0.608	0.642	0.579	0.805	0.758	0.525
S11	0.17	0.830	0.794	0.734	0.841	0.676	0.911
S13	0.46	0.828	0.782	0.717	0.907	0.632	0.932
S14	0.52	0.935	0.921	0.904	0.977	0.876	0.967
S15	0.51	0.635	0.657	0.584	0.787	0.731	0.584
S16	0.50	0.857	0.848	0.798	0.938	0.817	0.879
S17	0.49	0.892	0.889	0.855	0.958	0.877	0.901
Mean ± Std.	0.471 ± 0.125	0.784 ± 0.137	0.780 ± 0.097	0.729 ± 0.108	0.884 ± 0.096	0.768 ± 0.130	0.792 ± 0.241

**Table 9:** Statistical significance comparison of the Hybrid MST-Conformer versus baseline deep learning models across the 15 LOSO folds.

Comparison	Metric	Difference (Δ)	95% CI	Paired *t*-Test (*p*)	Wilcoxon (*p*)	Cohen’s *d*
Hybrid vs. BiLSTM	Bal. Acc.	+0.223	[+0.164,+0.280]	<0.001 ***	<0.001	+1.841
	F1	+0.303	[+0.199,+0.421]	<0.001 ***	<0.001	+1.340
	AUC	+0.249	[+0.181,+0.317]	<0.001 ***	<0.001	+1.825
	Accuracy	+0.236	[+0.151,+0.311]	<0.001 ***	<0.001	+1.444
	Sensitivity	+0.183	[−0.023,+0.391]	0.1209 ^ns^	0.1688	+0.426
	Specificity	+0.263	[+0.061,+0.452]	0.0226 *	0.0084	+0.662
Hybrid vs. CNN1D	Bal. Acc.	+0.221	[+0.175,+0.266]	<0.001 ***	<0.001	+2.357
	F1	+0.251	[+0.184,+0.317]	<0.001 ***	<0.001	+1.836
	AUC	+0.262	[+0.206,+0.320]	<0.001 ***	<0.001	+2.246
	Accuracy	+0.256	[+0.194,+0.311]	<0.001 ***	<0.001	+2.133
	Sensitivity	+0.103	[−0.036,+0.239]	0.1770 ^ns^	0.1688	+0.367
	Specificity	+0.338	[+0.200,+0.468]	<0.001 ***	<0.001	+1.248
Hybrid vs. CNN1D-MS	Bal. Acc.	+0.200	[+0.137,+0.260]	<0.001 ***	<0.001	+1.572
	F1	+0.225	[+0.151,+0.304]	<0.001 ***	<0.001	+1.425
	AUC	+0.243	[+0.181,+0.303]	<0.001 ***	<0.001	+1.966
	Accuracy	+0.238	[+0.147,+0.317]	<0.001 ***	<0.001	+1.375
	Sensitivity	+0.076	[−0.070,+0.226]	0.3443 ^ns^	0.4212	+0.253
	Specificity	+0.324	[+0.146,+0.488]	0.0029 **	0.0034	+0.929

*** p<0.001; ** p<0.01; * p<0.05; ^ns^ not significant (p≥0.05). Values report the paired difference (Δ) across the 15 LOSO folds. CI denotes the 95% confidence interval of the mean difference.

**Table 10:** Summary of the LOSO classification performance of the complete Hybrid MST-Conformer and the five ablation scenarios in Experiment 3 (mean ± std across 15 folds). The best mean value for each evaluation metric is shown in bold.

Configuration	Accuracy	Bal. Acc.	F1	AUC	Sensitivity	Specificity
Complete Hybrid	0.784±0.137	0.780±0.097	0.729±0.108	0.884±0.096	0.768±0.130	0.792±0.241
NoFeat (i)	0.715±0.152	0.701±0.124	0.606±0.181	0.851±0.106	0.656±0.264	0.747±0.284
FeaturesOnly (ii)	0.705±0.118	0.700±0.126	0.610±0.169	0.797±0.155	0.685±0.219	0.715±0.152
NoTCN (iii)	0.768±0.101	0.735±0.114	0.641±0.168	0.830±0.131	0.632±0.207	0.838±0.122
NoTransformer (iv)	0.752±0.098	0.748±0.058	0.681±0.062	0.872±0.061	0.738±0.138	0.759±0.203
Single-dilation TCN (v)	0.770±0.079	0.742±0.063	0.657±0.114	0.874±0.053	0.650±0.182	0.833±0.173

Bal. Acc.: Balanced Accuracy; Sensitivity: True Positive Rate (Activation); Specificity: True Negative Rate (Non-Activation).

**Table 11:** LOSO classification results obtained with the complete set of 27 ECG- and respiration-derived features using conventional classifiers (mean ± std across 15 folds). The best mean value for each evaluation metric is shown in bold.

Model	Accuracy	Bal. Acc.	F1	AUC	Sensitivity	Specificity
LDA	0.814±0.068	0.800±0.064	0.740±0.080	0.887±0.058	0.756±0.069	0.844±0.086
SVM	0.824±0.059	0.811±0.061	0.752±0.076	0.888±0.064	0.768±0.078	0.853±0.061
KNN	0.777±0.053	0.773±0.054	0.703±0.063	0.845±0.055	0.762±0.067	0.784±0.056
RF	0.824±0.053	0.807±0.053	0.748±0.069	0.894±0.055	0.751±0.070	0.863±0.063

LDA: Linear Discriminant Analysis; SVM: Support Vector Machine; KNN: k-Nearest Neighbors; RF: Random Forest; LOSO: Leave-One-Subject-Out; ECG: electrocardiogram; Bal. Acc.: Balanced Accuracy; Sensitivity: True Positive Rate (Activation); Specificity: True Negative Rate (Non-Activation).

**Table 12:** Subject-wise LOSO classification results obtained with the complete Hybrid MST-Conformer using the 27 physiological features.

Subject	Threshold	Accuracy	Bal. Acc.	F1	AUC	Sensitivity	Specificity
S2	0.48	0.850	0.827	0.773	0.927	0.755	0.898
S3	0.53	0.908	0.895	0.865	0.966	0.852	0.938
S4	0.55	0.818	0.774	0.703	0.844	0.637	0.911
S5	0.47	0.797	0.780	0.708	0.861	0.726	0.834
S6	0.54	0.848	0.821	0.767	0.893	0.735	0.906
S7	0.49	0.819	0.823	0.758	0.916	0.837	0.810
S8	0.49	0.874	0.881	0.832	0.959	0.904	0.858
S9	0.43	0.836	0.845	0.784	0.907	0.873	0.818
S10	0.50	0.688	0.689	0.613	0.779	0.694	0.684
S11	0.49	0.830	0.795	0.735	0.879	0.681	0.909
S13	0.40	0.833	0.834	0.776	0.926	0.837	0.830
S14	0.50	0.873	0.865	0.821	0.958	0.842	0.889
S15	0.35	0.685	0.734	0.666	0.841	0.896	0.571
S16	0.50	0.891	0.898	0.853	0.974	0.923	0.873
S17	0.48	0.829	0.844	0.793	0.937	0.900	0.789
Mean ± Std.	0.480 ± 0.051	0.825 ± 0.061	0.820 ± 0.056	0.763 ± 0.067	0.904 ± 0.053	0.806 ± 0.090	0.834 ± 0.093

**Table 13:** Summary of the LOSO classification performance of the complete Hybrid MST-Conformer and the five ablation scenarios in Experiment 4 using the complete set of 27 physiological features (mean ± std across 15 folds). The best mean value for each evaluation metric is shown in bold.

Configuration	Accuracy	Bal. Acc.	F1	AUC	Sensitivity	Specificity
Complete Hybrid	0.825±0.061	0.820±0.056	0.763±0.067	0.904±0.053	0.806±0.090	0.834±0.093
NoFeat (i)	0.561±0.073	0.578±0.064	0.493±0.083	0.612±0.083	0.638±0.196	0.518±0.165
FeaturesOnly (ii)	0.810±0.069	0.808±0.064	0.747±0.072	0.901±0.058	0.802±0.091	0.813±0.097
NoTCN (iii)	0.823±0.071	0.811±0.067	0.754±0.081	0.897±0.071	0.771±0.095	0.850±0.098
NoTransformer (iv)	0.806±0.085	0.801±0.064	0.743±0.075	0.898±0.063	0.788±0.085	0.815±0.131
Single-dilation TCN (v)	0.820±0.055	0.809±0.050	0.750±0.060	0.902±0.045	0.774±0.088	0.844±0.092

Bal. Acc.: Balanced Accuracy; Sensitivity: True Positive Rate (Activation); Specificity: True Negative Rate (Non-Activation).

**Table 14:** Comparison of the proposed framework with previous WESAD studies under Leave-One-Subject-Out (LOSO) cross-validation.

Study	Modalities	Task	Classifier	Accuracy	F1-Score/AUC
Schmidt et al. (2018) [23]	Chest (All 5 channels)	Stress vs. Non-Stress (baseline + amusement)	LDA/RF	0.930	0.910 (F1)
Bobade and Vani (2020) [40]	Multimodal (incl. ECG + RESP)	Stress vs. Non-Stress (baseline + amusement)	ANN	0.952	NR
Prajod and André (2022) [41]	ECG/HRV	Stress vs. Non-Stress (baseline + amusement + meditation)	Deep ECGNet	0.908	0.857 (F1)
Yang et al. (2025) [42]	Chest (RESP)	Normal (baseline + amusement) vs. Stress vs. Meditation	Stacking Ensemble	0.923	0.923 (F1)
Our Baseline (RF)	Chest (ECG + RESP)	Activation (stress + amusement) vs. Non-Activation (baseline + meditation)	Random Forest	0.824	0.748 (F1)/0.894 (AUC)
Our Baseline (SVM)	Chest (ECG + RESP)	Activation (stress + amusement) vs. Non-Activation (baseline + meditation)	Support Vector Machine	0.824	0.752 (F1)/0.888 (AUC)
Proposed Hybrid MST-Conformer	Chest (ECG + RESP)	Activation (stress + amusement) vs. Non-Activation (baseline + meditation)	Hybrid MST-Conformer	0.825	0.763 (F1)/0.904 (AUC)

The results should not be interpreted as a direct ranking across methods because the classification tasks and physiological modalities differ. In particular, the grouping of the original WESAD experimental conditions varies across studies.

## Data Availability

The WESAD dataset analyzed in this study is publicly available on the UC Irvine ML Repository (https://archive.ics.uci.edu/dataset/465/wesad+wearable+stress+and+affect+detection), accessed on 30 June 2026. The code needed for reproducing the experimentation can be found in https://github.com/asalazar-ehu/biosignal-ml-activation-detection (accessed on 30 June 2026), version 1.0.0. Besides, all the data for running that code can be found in the following Zenodo repository https://doi.org/10.5281/zenodo.23337725.

## Appendix A. Deep Learning Architecture Specifications

This appendix provides detailed layer-by-layer specifications of the baseline deep learning models implemented in this study.

### Appendix A.1. CNN1D Architecture

**Table A1:** Layer specifications for the CNN1D model.

Layer Type	Configuration	Output Shape
Input	20 s standardized ECG and RESP segments	(14,000, 2)
Conv1D	32 filters, kernel size 5, ReLU	(7000, 32)
Conv1D	64 filters, kernel size 5, ReLU	(3500, 64)
Conv1D	128 filters, kernel size 5, ReLU	(1750, 128)
AdaptiveAveragePooling1D	-	(128)
Dense	64 units, ReLU	(64)
Dropout	rate 0.5	(64)
Dense	1 unit, Sigmoid	(1)

### Appendix A.2. BiLSTM Architecture

**Table A2:** Layer specifications for the BiLSTM model.

Layer Type	Configuration	Output Shape
Input	20 s standardized ECG and RESP segments	(14,000, 2)
Conv1D	32 filters, kernel size 5, ReLU	(7000, 32)
Conv1D	64 filters, kernel size 5, ReLU	(3500, 64)
BiLSTM	32 units per direction	(3500, 64)
Dropout	rate 0.3	(3500, 64)
BiLSTM	16 units per direction	(3500, 32)
Dropout	rate 0.3	(3500, 32)
GlobalAveragePooling1D	-	(32)
Dense	32 units, ReLU	(32)
Dense	1 unit, Sigmoid	(1)

### Appendix A.3. CNN1D-MS Architecture

**Table A3:** Layer specifications for the CNN1D-MS model.

Layer Type	Configuration	Output Shape
Input	20 s standardized ECG and RESP segments	(14,000, 2)
Branch 1 (Fine Resolution)		
Conv1D	32 filters, kernel size 3, ReLU	(7000, 32)
Conv1D	64 filters, kernel size 3, ReLU	(3500, 64)
Conv1D	128 filters, kernel size 3, ReLU	(1750, 128)
AdaptiveAveragePooling1D	-	(128)
Branch 2 (Medium Resolution)		
Conv1D	32 filters, kernel size 7, ReLU	(7000, 32)
Conv1D	64 filters, kernel size 7, ReLU	(3500, 64)
Conv1D	128 filters, kernel size 7, ReLU	(1750, 128)
AdaptiveAveragePooling1D	-	(128)
Branch 3 (Coarse Resolution)		
Conv1D	32 filters, kernel size 15, ReLU	(7000, 32)
Conv1D	64 filters, kernel size 15, ReLU	(3500, 64)
Conv1D	128 filters, kernel size 15, ReLU	(1750, 128)
AdaptiveAveragePooling1D	-	(128)
Fusion		
Concatenate	branches 1, 2, 3	(384)
Dense	64 units, ReLU	(64)
Dropout	rate 0.5	(64)
Dense	1 unit, Sigmoid	(1)

## Appendix B. Algorithm Hyperparameters

### Appendix B.1. Hybrid MST-Conformer Hyperparameters and Architecture

**Table A4:** Training configuration used for the MST-Conformer experiments.

Parameter	Value
Random seed	42
Framework	PyTorch
Optimizer	AdamW
Initial learning rate	$4.38\times 10^{-4}$
Weight decay	$2.69\times 10^{-4}$
Learning-rate scheduler	OneCycleLR
Loss function	Focal Loss:
	$\alpha =1-\rho_{pos}$
	γ=1.356
	label smoothing = 0.012
Batch size	64
Maximum epochs	50
Early stopping patience	10 epochs
Training folds	LOSO (15 folds)
Hardware	Nvidia Tesla P40 GPU (3840 cores)
	24 GB memory
	346 GB/s bandwidth
Average training time per fold (min)	48.4±26.8
Total training time (h)	12.1

**Table A5:** Final MST-Conformer architecture and optimized hyperparameter values.

Parameter	Value
Input channels	2 (ECG and Respiration)
Handcrafted feature dimension	31
TCN filters per branch	16
Number of MST branches	3
Kernel size	3
Embedding dimension ($d_{model}$)	48
Number of transformer layers	1
Number of attention heads	2
Feed-forward dimension	128
Pooled sequence length	64
Feature branch hidden dimension	32
Dropout rate	0.337
Activation function	ReLU (TCN and MLP)
	GELU (Transformer)
Classification head	MLP (48+32→32→1)
Output classes	2

### Appendix B.2. Hyperparameters of the Conventional Machine Learning Classifiers

**Table A6:** Configuration of the conventional machine learning classifiers.

Classifier	Parameter Configuration
LDA	Discriminant type = pseudoLinear
SVM	RBF kernel, KernelScale = auto,
	Standardize = false
KNN	Number of neighbors = 5,
	Standardize = false
Random Forest	250 trees,
	MinLeafSize = 5,
	NumPredictorsToSample = all
Class balancing	Class weights inversely proportional to class frequencies
Random seed	rng(1,’twister’)
